# Exploration of Different Hypoxia Patterns and Construction of a Hypoxia-Related Gene Prognostic Index in Colorectal Cancer

**DOI:** 10.3389/fimmu.2022.853352

**Published:** 2022-05-30

**Authors:** Shuheng Bai, Ling Chen, Yanli Yan, Rong Li, Yun Zhou, Xuan Wang, Haojing Kang, Zhaode Feng, Guangzu Li, Shuling Zhou, Emmanuel Kwateng Drokow, Juan Ren

**Affiliations:** ^1^ Department of Radiotherapy, The First Affiliated Hospital of Xi’an Jiaotong University, Xi’an, China; ^2^ School of Medicine, Xian Jiaotong University, Xi’an, China; ^3^ Department of Chemotherapy, The First Affiliated Hospital of Xi’an Jiaotong University, Xi’an, China; ^4^ Department of Radiation Oncology, Zhengzhou University People’s Hospital & Henan Provincial People’s Hospital, Xi’an, China

**Keywords:** colorectal cancer, hypoxia, immune checkpoint inhibitors, tumor microenvironment (TME), hypoxia signaling pathway

## Abstract

**Introduction:**

Immune checkpoint inhibitor (ICI) therapy has been proven to be a highly efficacious treatment for colorectal adenocarcinoma (COAD). However, it is still unclear how to identify those who might benefit the most from ICI therapy. Hypoxia facilitates the progression of the tumor from different aspects, including proliferation, metabolism, angiogenesis, and migration, and improves resistance to ICI. Therefore, it is essential to conduct a comprehensive understanding of the influences of hypoxia in COAD and identify a biomarker for predicting the benefit of ICI.

**Methods:**

An unsupervised consensus clustering algorithm was used to identify distinct hypoxia-related patterns for COAD patients from TCGA and the GEO cohorts. The ssGSEA algorithm was then used to explore the different biological processes, KEGG pathways, and immune characteristics among distinct hypoxia-related clusters. Some hypoxia-related hub genes were then selected by weighted gene coexpression network analysis (WGCNA). Subsequently, univariate Cox regression analysis, multivariate Cox regression analysis, and least absolute shrinkage and selection operator (LASSO) regression were utilized to construct a hypoxia-related gene prognostic index (HRGPI). Finally, validation was also conducted for HRGPI in prognostic value, distinguishing hypoxia-related characteristics and benefits of ICI.

**Results:**

We identified four hypoxia-related clusters and found that different hypoxia response patterns induced different prognoses significantly. Again, we found different hypoxia response patterns presented distinct characteristics of biological processes, signaling pathways, and immune features. Severe hypoxia conditions promoted activation of some cancer-related signaling pathways, including Wnt, Notch, ECM-related pathways, and remodeled the tumor microenvironment of COAD, tending to present as an immune-excluded phenotype. Subsequently, we selected nine genes (ANO1, HOXC6, SLC2A4, VIP, CD1A, STC2, OLFM2, ATP6V1B1, HMCN2) to construct our HRGPI, which has shown an excellent prognostic value. Finally, we found that HRGPI has an advantage in distinguishing immune and molecular characteristics of hypoxia response patterns, and it could also be an excellent predictive indicator for clinical response to ICI therapy.

**Conclusion:**

Different hypoxia response patterns activate different signaling pathways, presenting distinct biological processes and immune features. HRGPI is an independent prognostic factor for COAD patients, and it could also be used as an excellent predictive indicator for clinical response to ICI therapy.

## Introduction

Colorectal cancer (CRC) is the third most common cancer and the second leading cause of cancer-related mortality, with a number of 1.8 million new cases and 881,000 deaths worldwide in 2018 (1). The most major pathological subgroup is adenocarcinoma, accounting for 90% of CRC. The 5-year survival rates for CRC patients with localized lesions and distant metastasis are 91% and 14%, respectively (2). Despite the recent advances in target therapy, including anti-VEFG or anti-EGFR, the prognosis of CRC patients with metastatic lesions remains poor. Hence, new effective therapies are urgently needed for this malignancy. Immune therapy, especially immune checkpoint inhibitors (ICIs), has been proven highly efficacious in CRC (3). ICIs can promote the anti-tumor immune activity of T cells by inhibiting a string of immune checkpoints, such as PD-1, PD-L1, and CTLA-4. To date, it is still unclear how to identify patients who will derive the most significant benefit from ICI (4). Microsatellite instability (MSI) and tumor mutational burden (TMB) have emerged as significant predictive markers for the efficacy of ICI, but the sensitivity and specificity are still urged to improve, and it is essential to construct new biomarkers (5).

Furthermore, a low response rate is still the most significant barrier to the effectiveness of ICI (6, 7). Also, many factors, especially the immune microenvironment (TME), can affect ICI effectiveness (8). TME is a complex and integral part of cancer, containing tumor cells, stromal cells, inflammatory cells, matrix fibers, metabolites, and cytokines.

Moreover, they interact with others and induce different outcomes, such as antitumor immune responses, immune suppression, or immune evasion, which are vital for tumor development (9). Hypoxia is a typical hallmark of TME in nearly all solid tumors, arising from the rapid and uncontrolled proliferation of tumors and insufficient blood supply (10). It promotes a more aggressive and metastatic phenotype mainly induced by HIFs, which are dimeric proteins consisting of an O_2_-sensitive subunit (HIF-1a, HIF-2a, or HIF-3a) and a scaffold b subunit (HIF-2b) (11). Under hypoxic conditions, HIFs bind with transcriptional coactivator and hypoxia response elements to increase the expression of a string of target genes, consequently regulating various biological processes, including proliferation, metabolism, angiogenesis, migration, and invasion (12–14). Moreover, hypoxia improves the resistance of tumor cells to chemotherapy, radiotherapy, and even immune therapy (15). It can regulate antitumor immune function by inhibiting T-cell proliferation and function, deactivating effector cytokine production, increasing the expression of some inhibitor receptors, and recruiting immunosuppressive cells (16–18). Therefore, it is essential to conduct a comprehensive understanding of the influence of hypoxia on colorectal cancer.

In this research, we first employed characteristics of different hypoxia patterns, including biological processes, signaling pathways, infiltration immune cells, and immune-related functions. We then constructed hypoxia-related gene prognostic signatures (HRGPI) and characterized their molecular and immune features. Furthermore, we detected its predictive ability for patients with COAD who received immune checkpoint inhibitors (ICI).

## Methods

### Data Acquiring and Processing

All colorectal adenocarcinoma (COAD) data in this research were obtained from The Cancer Genome Atlas (TCGA) and the Gene Expression Omnibus (GEO) datasets. RNA-seq data (level 3, count workflow) and corresponding clinical data of 514 COAD samples, including 473 tumor samples and 41 adjacent normal samples, were obtained from TCGA dataset (https://portal.gdc.cancer.gov/). Microarray data and corresponding clinical data of 232 COAD samples (GSE17536 and GSE17537), which were all tumor samples, were obtained from the GEO dataset (https://www.ncbi.nlm.nih.gov/gds). The raw microarray data from the GEO dataset were processed *via* background correction, log2 transformation, quantile normalization, and annotation in R software using the package Affy. The RNA-seq data of TCGA were transformed into TPM value from the count value. The meta-cohort data were a combination of TCGA and the GEO data, and the ComBat algorithm of the SVA package was utilized to decrease the possibility of batch effects of nonbiological technical biases from each dataset. The corresponding clinical data mainly included age, gender, tumor stage, and overall survival time, as shown in **Supplementary Tables S1 and S2**.

### Clustering for Different Hypoxia-Related Patterns

The hypoxia gene set was downloaded from the Molecular Signatures Database (MSigDB) of the Broad Institute (https://www.gsea-msigdb.org/gsea/msigdb/index.jsp). The package ConsensusClusterPlus was an unsupervised consensus clustering method, which was utilized to identify distinct hypoxia patterns based on these hypoxia genes and determine the number of clusters in the meta-cohort data.

Furthermore, to visualize the result of hypoxia-related clusters, algorithms of t-distributed stochastic neighbor embedding (t-SNE) and principal component analysis (PCA) were utilized to conduct dimension reduction analysis for these hypoxia gene signatures of tumor samples. Moreover, these two algorithms were called from the Rt-SNE and PCA packages, respectively, in R software.

### Identification of Hypoxia-Related Hub Genes

According to the consensus clustering results, distinct hypoxia-related patterns based on the expression of hypoxia response-related genes were identified. Weighted gene coexpression network analysis (WGCNA) was performed to select hypoxia-related genes: (1) The similarity matrix was constructed using the expression data by calculating the Pearson correlation coefficient. (2) The similarity matrix was transformed into an adjacency matrix, and a soft-threshold *β* = 5 was adopted. (3) The adjacency matrix was transformed into a topological overlap matrix (TOM). 1-TOM was used as the distance to cluster the genes, and then the dynamic pruning tree was built to identify the modules. (4) The merging threshold function was set at 0.25 to identify modules. (5) The correlation between the hypoxia clusters and these modules was calculated to select the most significant module.

To further screen these hypoxia-related genes, the different expression genes (DEGs) between tumor samples and normal samples were overlapped with these hypoxia-related genes, and finally hypoxia-related hub genes were obtained. The package Limma in R was adopted in this procedure.

### Construction and Validation of Hypoxia-Related Gene Prognostic Index

A string of hypoxia-related hub genes was obtained above. Univariate Cox regression analysis and least absolute shrinkage and selection operator (LASSO) regression were then conducted for these selected genes *via* the Survival and Glmnet package. Afterward, multivariate Cox regression analyses were utilized to construct a prognostic model called HRGPI. The HRGPI score was calculated using the following formula: HRGPI score = $\sum_{i=1}^{n}\text{coef}\times \text{gene}$ . A Kaplan–Meier survival analysis was also carried out to assess the difference in survival between high- and low-score groups using the Survival package.

Additionally, a nomogram was built using the RMS R package, based on the HRGPI calculated above and some clinical features. Furthermore, the calibration plot was applied to explore the nomogram’s calibration and discrimination by utilizing the RMS R package. A string of comprehensive indexes, concluding the time-dependent receiver operating characteristic (ROC) curve and the decision curve analysis (DCA), was then calculated to evaluate the prediction efficiency and clinical usefulness for HRGPI, this nomogram, and stage, by utilizing the survivalROC and the ggDCA in R.

### Function and Pathway Enrichment Analysis

The single-sample gene set enrichment analysis (ssGSEA) algorithm was performed to identify the difference in biological process and the Kyoto Encyclopedia of Genes and Genomes (KEGG) pathways between distinct hypoxia-related clusters using the GSVA package in R. The referenced gene sets of the KEGG pathways and hallmark gene sets were used biological function annotations, which were downloaded from MSigDB. The GSEA algorithm was also utilized to understand the difference in functions and pathways between the HRGPI-high and HRGPI-low groups by applying the clusterProfiler R package.

### Comprehensive Analysis of Molecular and Immune Characteristics and ICB Treatment Effect

In this research, the estimate algorithm was utilized to evaluate the overall infiltration of immune cells and stromal cells (19). The ssGSEA algorithm was also utilized to evaluate immune-related functions and infiltration immune cells in each sample. The marker gene sets of immune-related functions and infiltration immune cell types were obtained from the studies of He et al. (20) and Charoentong et al. (21). To further define the molecular characteristics between HRGPI-high and HRGPI-low groups, the consensus molecular subtype (CMS) classification of colorectal cancer was obtained from Guinney et al. (22). Again, the different expression levels of the main immune checkpoints, including PD-L1 and CTLA-4, were compared among different hypoxia clusters or HRGPL groups.

The tumor immune dysfunction and exclusion (TIDE) algorithm and IMvigor210 (A Study of Atezolizumab in Participants with Locally Advanced or Metastatic Urothelial Bladder Cancer; mUC) dataset were also used to explore the prognostic of HRGPI in patients after immunotherapy. TIDE is a computational framework developed to evaluate the potential of tumor immune escape from the gene expression profiles of cancer samples, which can predict the outcome of cancer patients treated with anti-PD1 or anti-CTLA4 more accurately than other biomarkers (23). Moreover, the TIDE score was calculated online (http://tide.dfci.harvard.edu/) in this research. The IMvigor210 was a dataset that contained patients with mUC receiving PD-L1 inhibitor atezolizumab (24), and it was utilized in this study to validate the effectiveness of HRGPI. The raw transcriptomic and clinical data were obtained using the package IMvigor in R.

### Statistical Analysis

The Wilcoxon test and Kruskal test were performed to compare continuous variables between two groups and more than two groups, respectively. Categorical data were compared using the Chi-square test and Fisher test. The Kaplan–Meier survival curves were depicted, and the significant differences between survival curves were determined with the log-rank test. All statistical analyses in this research were performed with R software 3.6.1. Furthermore, a two-sided *p*-value <0.05 was considered significant.

## Results

### Identification of Hypoxia-Related Clusters

**Figure 1:**
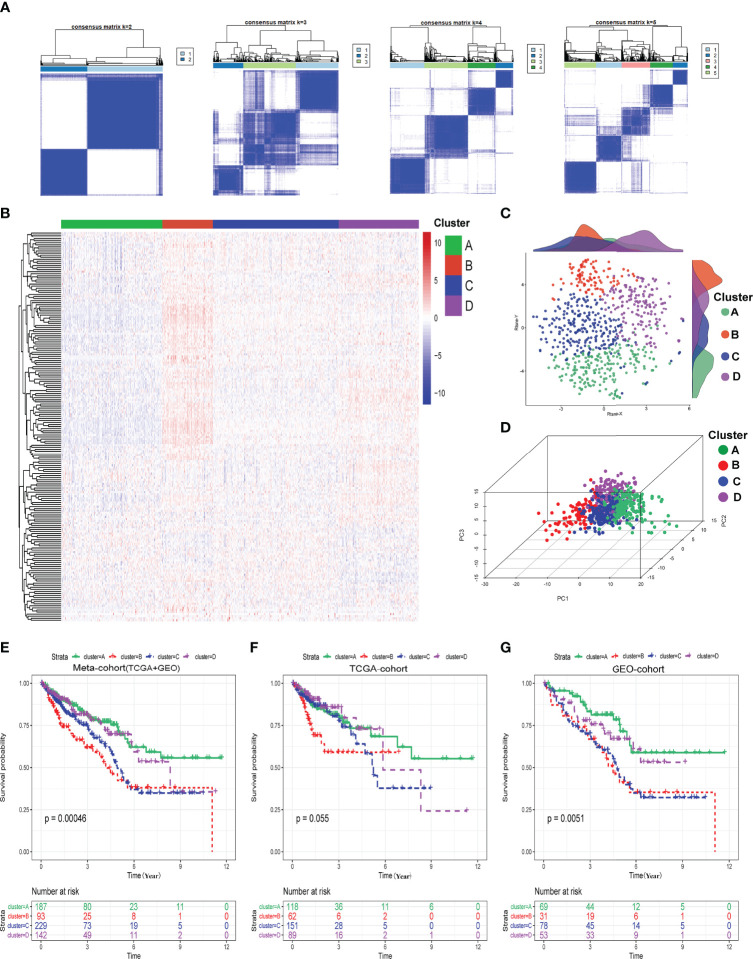
Identification of hypoxia-related clusters. **(A)** Determination of four distinct hypoxia-related clusters by ConsensusClusterPlus. **(B)** Heatmap about the expressions of hypoxia genes among four clusters. **(C)** Dot plot for these four clusters by t-SNE algorithms. **(D)** Dot plot for these four clusters by PCA algorithms. **(E–G)** Survival analysis (Kaplan–Meier) in meta-cohort, TCGA cohort, and GEO cohort.

We determined the optimal cluster number and identified four distinct hypoxia-related clusters in the meta-cohort by utilizing the ConsensusClusterPlus algorithm, as shown in **Figure 1A**. The four hypoxia-related clusters were labeled A to D, containing 187, 93, 229, and 142 patients. Unsupervised hierarchical clustering showed that hypoxia genes were differentially expressed among these four clusters (**Figure 1B**). Moreover, we could find that the discrimination of our clusters was excellent by visualizing the results of t-SNE and PCA algorithms, as shown in **Figures 1C, D**. Furthermore, we conducted Kaplan–Meier (K-M) survival analysis in the meta-cohort, TCGA cohort, and GEO cohort in turn and demonstrated that prognoses were statistically different among these four clusters (**Figures 1E–G**). Moreover, we also discovered that cluster A exhibited the best overall survival (OS) time and progression-free survival (PFS) time among these four clusters; however, cluster B presented the worst (**Figures 1E–G**; **Supplementary Figures S1**–**S3**, and **S4A–J**).

### Biological Processes and Immune Characteristics of Different Hypoxia-Related Clusters

**Figure 2:**
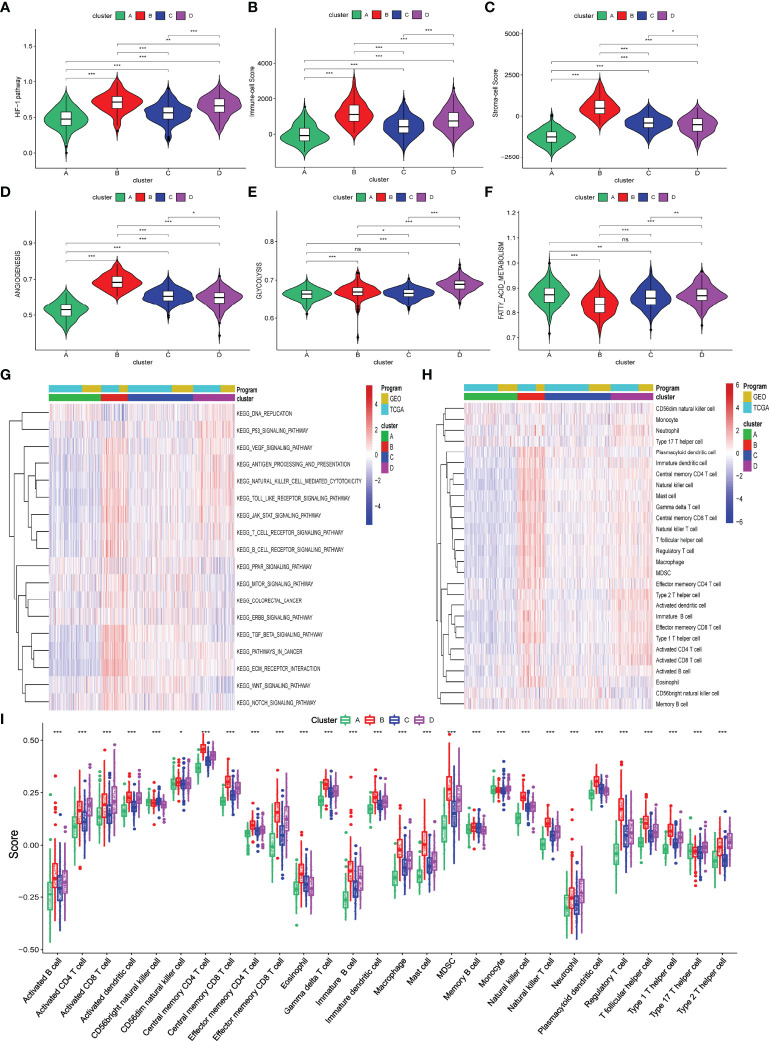
Biological processes and immune infiltration in different hypoxia-related clusters. **(A–F)** Six biological processes were different among these four hypoxia-related clusters. **(G)** Heatmap about KEGG pathways among four hypoxia-related clusters. **(H**, **I)** Infiltration immune cells were significantly distinct in these four clusters. (*p*>0.05 ns; *p* < 0.05 *; *p* < 0.01 **; *p* < 0.001 ***).

Hypoxia status can activate the HIF-1 pathway to affect cancer cells’ angiogenesis and metabolism processes and the infiltration of immune cells into TME. So, we first briefly explored these biological processes among these four hypoxia-related clusters using the ssGSEA algorithm. As shown in **Figures 2A–F** and **Supplementary Table S3**, levels of activation of these biological processes were different among these four hypoxia-related clusters. Moreover, cluster B was markedly activated in the HIF-1 pathway, angiogenesis process, immune cell infiltration, and stromal cell infiltration, while cluster A had the lowest activation. Meanwhile, clusters A and B activated glycolysis and fatty acid metabolism differently. We also explored a string of tumor-related pathways by performing ssGSEA for KEGG pathways. In **Figure 2G** and **Supplementary Table S4**, the immune and carcinogenic pathways were mainly activated in cluster B, namely the VEGF signaling pathway, antigen presentation pathway, T/B cell receptor pathway, TGF-beta signaling pathway, Wnt signaling pathway, Notch signaling pathway, extracellular matrix (ECM) signaling pathway, and colorectal cancer signaling pathway.

**Figure 3:**
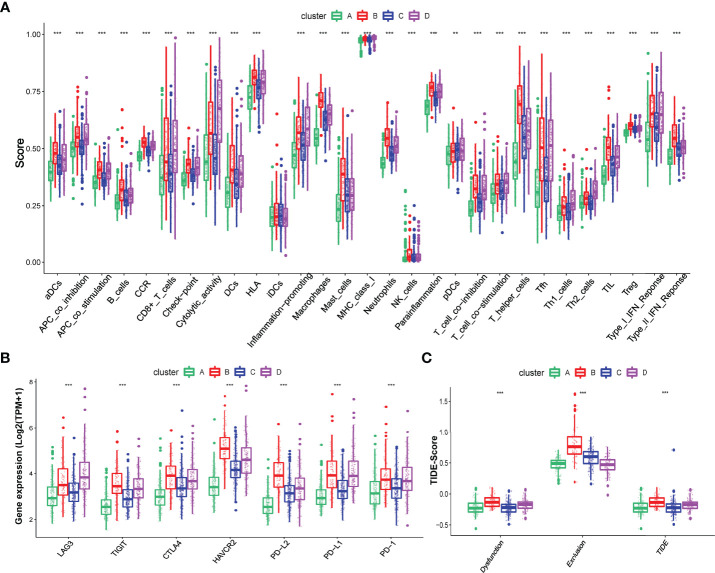
Different immune characteristics in these four hypoxia clusters. **(A)** Different activation of immune functions among these four hypoxia clusters. **(B)** Different expressions of immune checkpoints among these four hypoxia clusters. **(C)** Different TIDE scores among these four hypoxia clusters. (*p* < 0.05 *; *p* < 0.01 **; *p* < 0.001 ***).

To explore the difference in immune characteristics among these four hypoxia-related clusters, we subsequently analyzed the composition of immune cells and activation of immune functions in different clusters. The infiltration immune cells were significantly distinct in these clusters, and cluster B was characterized by high infiltration immune cells while cluster A was the opposite (**Figures 2H, I**; **Supplementary Table S5**). Interestingly, cluster B significantly increased not only in the infiltration of cells that promote immune function, such as activated B cells, memory CD4^+^ T cell, and effector memory CD8^+^ T cell but also in the infiltration of cells that inhibit immune function, containing myeloid-derived suppressor cells (MDSC) and regulator T cells (Treg). We also found that activation of immune functions was different in these four hypoxia clusters, as shown in **Figure 3A**. Cluster B presented a marked activation in anticancer immune function such as antigen-presenting cell (APC) stimulation, CD8^+^ T cells, and NK cells; however, the function of immune inhibitions was also activated in cluster B, such as APC inhibition, checkpoints, Treg cell, and T-cell inhibition, which accorded with the result of infiltration immune cells.

We also explored the different expressions of immune checkpoints and the benefits of immune therapy among these four clusters. As **Figure 3B** shows, patients in cluster B had a higher expression of checkpoints, including PD-L1 and PD-1, indicating a tendency for immune evasion. In addition, samples in cluster B had the highest TIDE score, representing a higher potential for immune evasion and suggesting a fewer benefit from ICI therapy (**Figure 3C**).

### Construction and Validation of Hypoxia-Related Gene Signature Index

**Figure 4:**
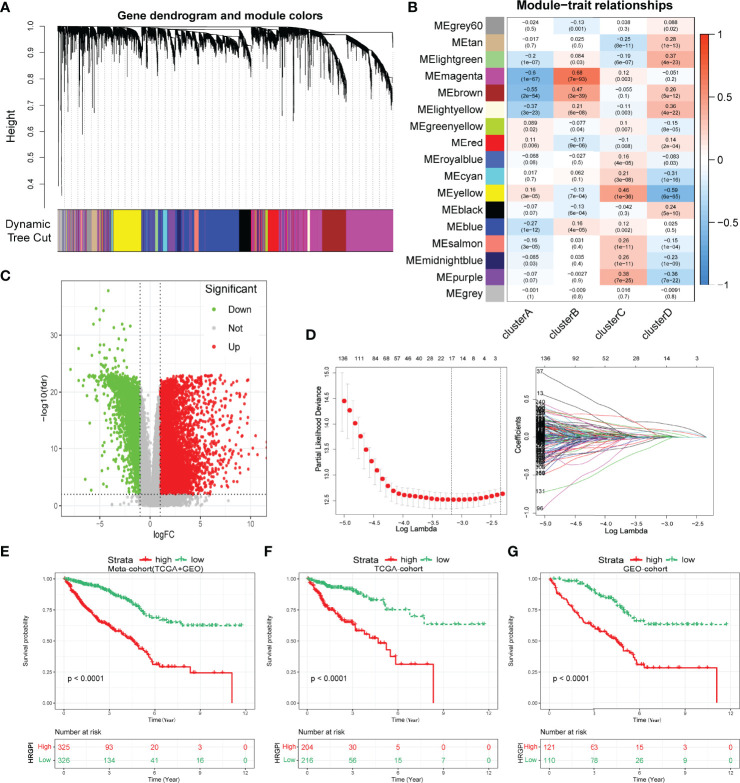
Construction of hypoxia-related gene signature index (HRGPI). **(A)** Identification of 17 modules by setting the merging threshold function at 0.25. **(B)** Correlation coefficient between a module and sample feature for each module. **(C)** Volcano plot about DEGs between tumor and normal samples. **(D)** Determination of the number of factors by the LASSO analysis. **(E–G)** Kaplan–Meier plots of OS for patients in the HRGPI-high and HRGPI-low groups.

Biological processes and immune characteristics were distinct, as shown above. Therefore, to identify some hypoxia-related genes, we first conducted a WGCNA analysis and identified 17 modules by setting the merging threshold function at 0.25, as shown in **Figure 4A**. According to the Pearson correlation coefficient between a module and sample feature for each module, the magenta module closely correlated with clusters A and B (**Figure 4B**). These genes in the magenta module were selected as hypoxia-related genes and then overlapped with the differentially expressed genes (DEGs) between tumor and normal samples to obtain hypoxia-related hub genes (**Figure 4C**; **Supplementary Tables S6 **and** S7**).

Subsequently, *via* utilizing univariate Cox regression, multivariable Cox regression, and LASSO regression, we finally constructed our HRGPI prognostic model, containing a total of nine signature genes (ANO1, HOXC6, SLC2A4, VIP, CD1A, STC2, OLFM2, ATP6V1B1, HMCN2), as shown in **Figure 4D** and **Supplementary Tables S8 **and** S9**. Taking the median HRGPI as the cutoff value, we conducted K-M survival analysis for OS and PFS and found that a higher HRGPI score indicated a worse prognosis (**Figures 4E–G**; **Supplementary Figure S4H**). We also found that the patients in cluster A had a higher HRGPI score than samples in cluster B, which was consistent with our results above (**Supplementary Figure S5**).

**Figure 5:**
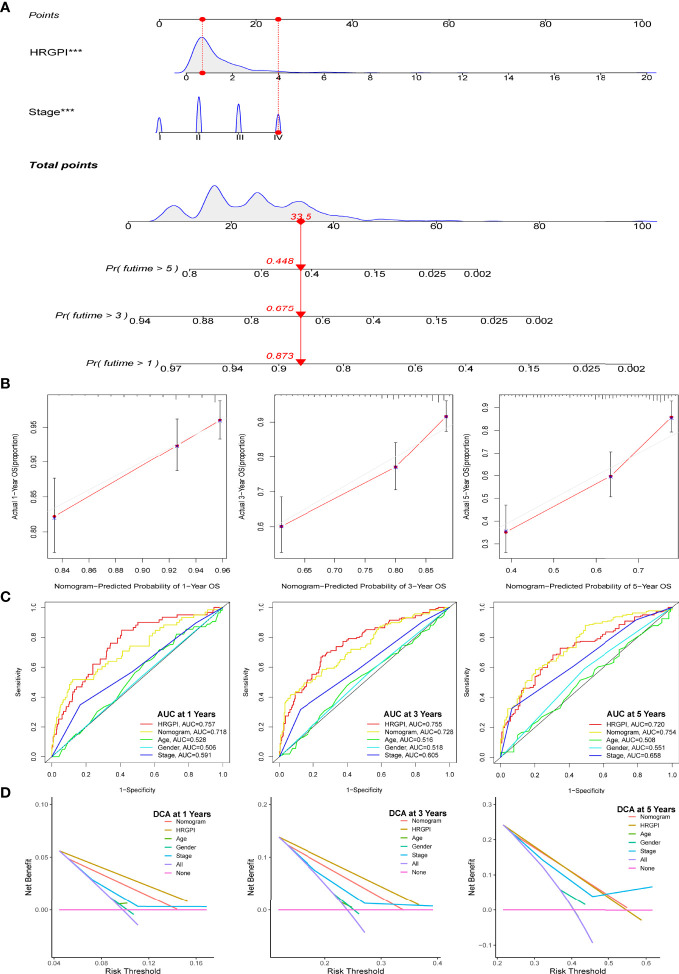
Construction and validation of a nomogram containing HRGPI. **(A)** A nomogram to predict the 1-, 3-, and 5-year OS. **(B)** Calibration curves for the nomogram model. **(C)** ROC curves for the 1-, 3-, and 5-year OS. **(D)** DCA curves for the 1-, 3-, and 5-year OS. (*p* < 0.05 *; *p* < 0.01 **; *p* < 0.001 ***).

Moreover, we enrolled HRGPI and clinical stage, age, and gender into a multivariate Cox regression analysis to determine whether the HRGPI was a clinically independent prognostic factor for colorectal patients. Furthermore, a nomogram containing independent factors was constructed, as shown in **Figure 5A**. We then portrayed the corresponding calibration plots in 1, 3, and 5 years, and the performance of the nomogram was excellent (**Figure 5B**).

Thereafter, we conduct a series of analyses to verify the effectiveness and sensitivity of our HRGPI. A ROC analysis was conducted first. The area under the curve (AUC) values of HRGPI for 1-, 3-, and 5-year OS were 0.757, 0.7555, and 0.72, respectively. Moreover, compared with other clinical features and the nomogram, the ROC analysis indicated that HRGPI was better than other features (**Figure 5C**). We also carried out the DCA analysis to determine the clinical usefulness of HRGPI by quantifying the net benefits at different threshold probabilities. As shown in **Figure 5D**, the character of HRGPI had excellent clinical effectiveness compared with the clinical stage.

### Biological Process and Immune characteristics of different HRGPI groups

**Figure 6:**
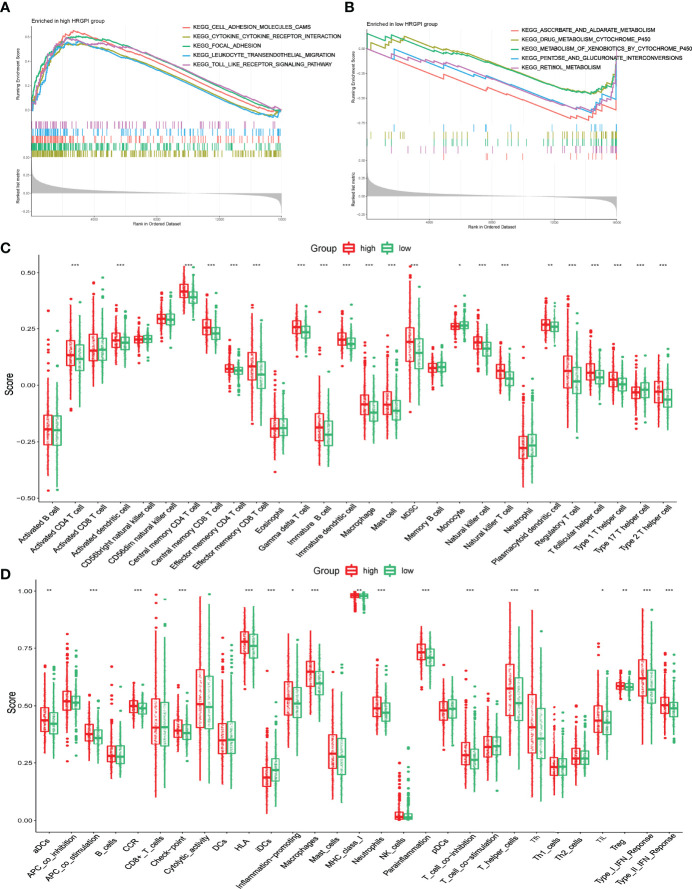
Biological process and immune characteristics of different HRGPI groups **(A, B)** Distinct KEGG pathways enrich in different HRGPI groups by GSEA algorithm. **(C)** Infiltration immune cells were distinct in different HRGPI groups. **(D)** Different activation of immune functions in different HRGPI groups. (*p* < 0.05 *; *p* < 0.01 **; *p* < 0.001 ***).

GSEA was utilized to determine the KEGG pathways enriched in different HRGPI groups. The gene sets of HRGPI-high samples were mainly enriched in cytokine–cytokine receptor interaction, cell adhesion molecules, leukocyte transendothelial migration, and Toll-like receptor signaling pathways (**Figure 6A**). Moreover, the gene sets of HRGPI-low samples were principally enriched in ascorbate metabolism, drug metabolism cytochrome P450, and retinol metabolism signaling pathways (**Figure 6B**). Detailed results of the GSEA are presented in **Supplementary Table S10**.

We then explored these biological processes briefly between the HRGPI-high and HRGPI-low groups. As shown in **Supplementary Figure S6**, the HRGPI-high group markedly activated in the HIF-1 pathway, angiogenesis process, immune cell infiltration, and stromal cell infiltration, while HRGPI-low groups had a fewer activation. Meanwhile, glycolysis and fatty acid metabolism were also activated differently between these two subgroups. Subsequently, we explored the different compositions of infiltration immune cells between the HRGPI-high and HRGPI-low groups. As shown in **Figure 6C**, we found that some immune promoter cells, such as activated CD4^+^ T cell, activated dendritic cell, effector memory CD8^+^ T cell, and natural killer cell, were more abounded in the HRGPR-high samples, while cells (including Treg cells and MDSC) that present a function of immune inhibition also abounded in the HRGPI-high samples. The phenomenon was striking and conformed with our results about the infiltration of immune cells in hypoxia cluster A. Moreover, we found that activation of immune functions was different in different HRGPI groups, as shown in **Figure 6D**. HRGPI-high samples presented a marked activation in the function of immune inhibitions, such as checkpoints, Treg cell, and T-cell inhibition, which was consistent with the composition of infiltration immune cells mentioned above.

### Molecular Subtypes of Different HRGPI Groups

**Figure 7:**
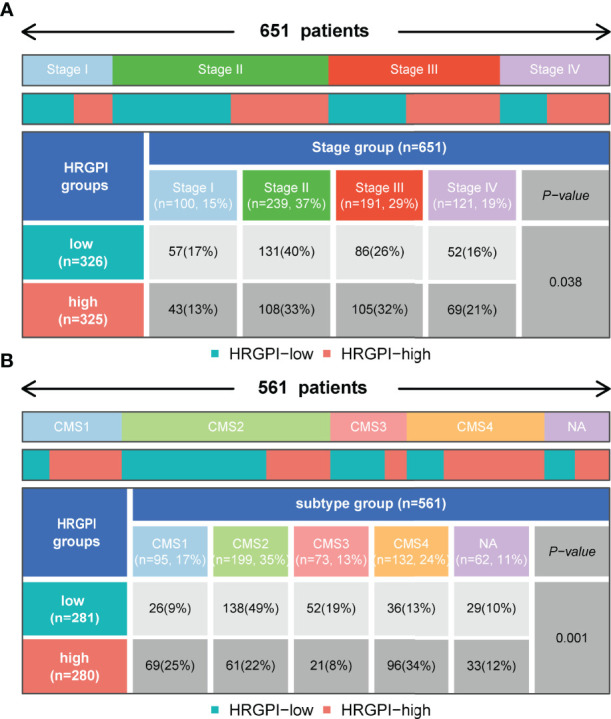
Distribution of clinical-stage, molecular and immune subtypes in different HRGPI groups. **(A)** Heatmap and table showing the distribution of clinical stage in different HRGPI groups. **(B)** Heatmap and table showing the distribution of CMS subtypes in different HRGPI groups.

We employed the distribution of clinical stage in different HRGPI groups. As shown in **Figure 7A**, the HRGPI-low group comprised 17% stage I patients, 40% stage II patients, 26% stage III patients, and 16% stage IV patients, while the HRGPI-high group comprised 13% stage I patients, 33% stage II patients, 32% stage III patients, and 21% stage IV patients. Moreover, there were more patients with advanced stages in the HRGPI-high group than in the HRGPI-low group.

The international colorectal cancer subtyping consortium analyzed six independent classification systems and proposed a robust classification system named consensus molecular subtypes (CMS): CMS1 (MSI immune), characterized by hypermutated, microsatellite unstable, strong immune activation; CMS2 (canonical), chromosomally unstable, marked WNT and MYC signaling activation; CMS3 (metabolic), evident metabolic dysregulation; and CMS4 (mesenchymal), prominent transforming growth factor β activation, stromal invasion, and angiogenesis (22). We then focused on the distribution of the CMS molecular subtypes in the different HRGPI groups. The proportions of CMS subtypes in the two groups were statistically different, and there were more patients classified into CMS1 and CMS4 and fewer samples classified into CMS2 and CMS3 in the HRGPI-high group than in the HRGPI-low group (**Figure 7B**; **Supplementary Table S11**).

### The Benefits of Immune Therapy in Different HRGPI Groups

**Figure 8:**
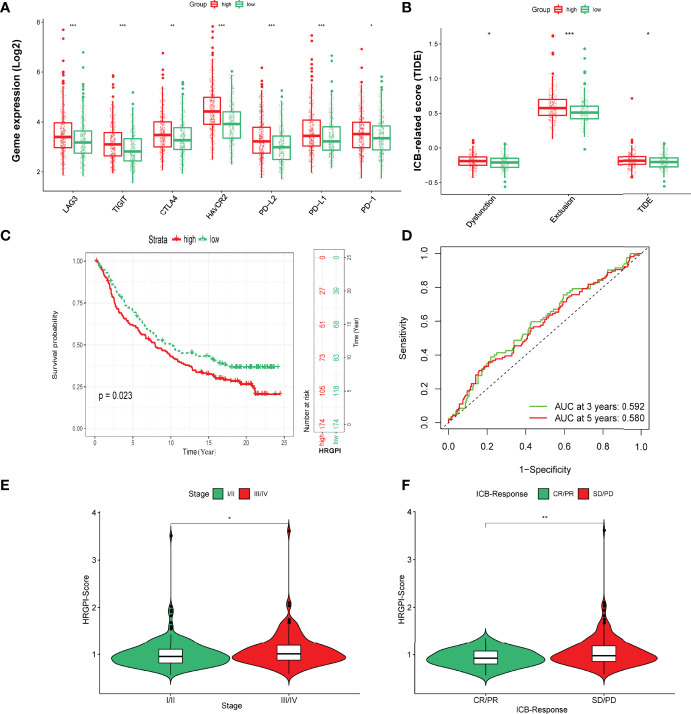
The prognostic value of HRGPI in patients with ICI therapy. **(A)** Different expressions of immune checkpoints in different HRGPI groups. **(B)** Different TIDE scores in different HRGPI groups. **(C)** Kaplan–Meier plots of the HRGPI subgroups in the mUC cohort. **(D)** ROC curves of HRGPI for the 1-, 3-, and 5-year OS of patients in the mUC cohort. **(E)** Patients with advanced stages in the mUC cohort have a higher HRGPI score. **(F)** Patients with SD/PD status after ICI treatment in the mUC cohort have a higher HRGPI score. (*p* < 0.05 *; *p* < 0.01 **; *p* < 0.001 ***).

Finally, we tried to assess the potential clinical efficacy of immunotherapy in different HRGPI subgroups. Firstly, the expression levels of some immune checkpoints were compared between the two groups. As shown in **Figure 8A**, the expression of PD-L1, PD-1, CTLA-4, HAVCR2, LAG3, and TIGIT was higher in the HRGPI-high group than in the HRGPI-low group. We then utilized the TIDE algorithm to evaluate the efficacy of immunotherapy. Our results showed that the HRGPI-high group had higher TIDE scores than the HRGPI-low group, which indicated a higher potential for immune evasion and a fewer benefit from ICI therapy in the HRGPI-high group (**Figure 8B**). Furthermore, the scores of T-cell dysfunction and T-cell exclusion were significantly different in these two groups. Moreover, IMvigor210 (mUC) cohort was also used to verify ICI benefits in the two groups. K-M survival curves revealed that samples with a low HRGPI score exhibited significant benefits and an apparent prolonged OS compared with the HRGPI-high group (**Figure 8C**). However, the effectiveness and sensitivity of HRGPI in the IMvigor210 cohort need to be improved, as AUC values for 3- and 5-year OS were 0.592 and 0.58, respectively (**Figure 8D**). We also found that the patients with advanced stages had a higher HRGPI score, and those with a CR/PR status after ICI treatment had a lower HRGPI score (**Figures 8E, F**). It conformed with the TIDE results above and indicated that HRGPI could effectively assess the clinical efficacy of immunotherapy.

## Discussion

Studies have demonstrated that the TME can be reprogrammed under hypoxia status to induce abnormal angiogenesis, desmoplasia, and inflammation, all of which contribute to tumor progression and therapeutic resistance (15). The hypoxia-induced biological functions are mediated by a string of signaling pathways, including the Notch pathway, the Wnt pathway, and especially the HIF-1 signaling pathway (25). Hypoxia leads to immune tolerance by inhibiting APC recognition and dampening the capability of effector immune cell-mediated elimination of tumor (26). Therefore, it is essential to comprehensively understand the underlying mechanism of the immune response of anti-tumor by exploring the role of distinct hypoxia response patterns in biological processes and the TME immune characteristics.

In this present study, four distinct hypoxia-related clusters were first identified. These four clusters exhibited significant differences in OS, PFS, biological processes, KEGG signaling pathways, and immune characteristics. The patients in cluster B had the worst prognosis (OS and PFS), compared to patients in cluster A. Moreover, we also found cluster B featured high-level activation of the HIF-1 pathway, angiogenesis process, and glycolysis process; however, opposite characteristics were seen in cluster A. This phenomenon indicated that cluster B had a more obvious hypoxia status than cluster A. Tumor hypoxia induces HIF-1 overexpression to regulate a string of biological processes (27). Under hypoxia stress, the inhibitory hydroxylation of HIF-1α is reduced, leading to the stability and translocation of HIF-1α to the nucleus, where it heterodimerizes with HIF-1β, finally inducing some overexpression of target genes (28). A study also found that excessed HIF-1 could activate VEGF transcription, thereby stimulating the process of angiogenesis and contributing to tumor progression (29). Furthermore, the metabolism process of tumor cells also changes when faced with hypoxia conditions. HIF-1 promotes glucose uptake by activating the transcription of transporters GLUT1 and GLUT3 and enhances glycolysis by upregulating most of the glycolytic enzymes, including ENO1, PGK1, and PKM2, to improve anaerobic energy production (30, 31). For lipid metabolism, hypoxia mainly enhances lipogenesis and relied on HIF-1-related modulation of proteins involved in fatty acid (FA) uptake, synthesis, storage, and usage, and simultaneously the catabolism of FA is impaired (30). Researchers have demonstrated that hypoxia can stimulate tumor proliferation and metastasis *via* activating Wnt, Notch, and ECM-related pathways (13, 32, 33). Consistent with our results, most carcinogenic pathways were mainly activated in cluster B, including the Wnt signaling pathway, Notch signaling pathway, and ECM signaling pathway.

Hypoxia facilitates malignant tumor escape from immune surveillance by promoting innate and adaptive immune evasion (27). In the innate immune part, HIF-1 can activate downstream target genes and immunosuppressive molecules (as COX-2, HIG-2), impairing the differentiation, immune function, and maturation of NK cells and DCs (34–36). In the adaptive immune part, the enhanced HIF-1 not only facilitates the recruitment and stimulation of immune-suppressive cells, including Treg and MDSC, but also improves the secretion of TH2-type cytokines and impairs the production of TH1-type cytokines, resulting in the inhibition of the antitumor immune function of CD8^+^ T cells and the activity of APCs (18, 36, 37). Our research found that the immune characteristics were distinct among the four hypoxia patterns. As analyzed above, cluster B was indicated to have the most apparent hypoxia status. Moreover, we found it was characterized by high infiltration immune cells and stromal cells, while cluster A was the opposite. Interestingly, we found that not only MDSC and Treg but also B cells, memory CD4^+^ T cells, and CD8^+^ T cells, all presented a high infiltration level in cluster B. Therefore, we presume the hypoxia TME of COAD was complex and tended to be labeled as an immune-excluded phenotype. The presence of abundant immune cells characterizes this phenotype; however, the immune cells do not penetrate the parenchyma of tumors but are retained in the stroma surrounding the nests of tumor cells (38). In the aftermath treatment with anti-PD-L1/PD-1 agents, stroma-associated T cells are shown as activation and proliferation but not infiltration. This phenomenon was also consistent with our results regarding immune functions. Also, we found that patients in cluster B had a higher expression of checkpoints, including PD-L1, PD-1, and CTLA4. Many studies identified that immune checkpoints are negative regulatory molecules in the immune system (38, 39), and once activated, these molecules can inhibit immune cell functions and impair an effective antitumor immune response (27, 40). In addition, patients in cluster B had the highest TIDE score, T-cell exclusion score, and T-cell dysfunction score, which suggested a fewer benefit from ICI therapy and conformed to the results of infiltration immune cells and immune functions. According to these hypoxia-related characteristics of COAD, it may be better to focus on stroma (such as ECM-related target sites) simultaneously when conducting ICI therapy for COAD patients. ECM plays a vital role in the development and progression of cancer, influencing the regulation of immune cell migration and function (41). Peter et al. found that ECM is an obstacle for immune cells to make direct contact with adjacent tumor cells and thus limits colorectal tumor therapy (42). Studies found that losartan impairs collagen and hyaluronan deposition in various malignancies, reducing hypoxia status and improving tissue perfusion (43, 44). Treatment for ECM will most likely improve tumor immunity by promoting T-cell migration and inhibiting a myelosuppressive phenotype; however, more clinical trials are needed to demonstrate the therapeutic effectiveness of immune therapies (41).

Based on the distinct characteristics among the four hypoxia-related clusters, we then identified nine signature genes and constructed a hypoxia-related prognostic index named HRGPI by utilizing a string of algorithms such as WGCNA and LASSO regression analysis. The HRGPI proved to be a valid prognostic hypoxia-related biomarker for COAD, with better survival in HRGPI-low patients and worse survival in HRGPI-high patients. Meanwhile, the effectiveness and sensitivity of our HRGPI were also outstanding compared with clinical stage, age, and even a nomogram that contained HRGPI and stage. Zhang et al. constructed a prognostic hypoxia model based on five genes. However, the AUC values for 1, 3, and 5 years in their internal validation group were only 0.614, 0.585, and 0.514, respectively (45). Yang et al. also built a hypoxia-related model using four miRNAs, and the AUC values for 3 and 5 years were 0.711 and 0.737, respectively, which are as good as our HRGPI (46).

Our HRGPI consisted of nine genes, ANO1, HOXC6, SLC2A4, VIP, CD1A, STC2, OLFM2, ATP6V1B1, and HMCN2. From our best research, HOXC6, SLC2A4, VIP, CD1A, STC2, and OLFM2 are related to cancer progression. Homeobox C6 (HOXC6) is a transcription factor that plays a vital role in several cancers, including colorectal cancer (47, 48). The overexpression of HOXC6 can promote the migration and invasion of colon cancer cells by inducing epithelial-mesenchymal transition (EMT) *via* activating the Wnt/β-catenin signaling pathway (49). Solute carrier family-2-member-4-gene (Slc2a4) encodes GLUT4, which functions as an insulin-regulated facilitative glucose transporter and has been identified as a promising therapeutic target for cancer (50). Vasoactive intestinal peptide (VIP) influences solid tumor angiogenesis (51). Collado et al. found that VIP does not stimulate HIF-1 mRNA expression but increases the translocation of HIF-1 from the cytoplasm to the nucleus (52). CD1A is a member of the CD1 family, which mediates MHC-independent pathways for antigen presentation and T-cell activation (53). Stanniocalcin 2 (STC2) gene is a glycoprotein hormone involved in glutamine or glucose deprivation, and it also was upregulated under hypoxia status. It can contribute to tumor cells’ adaptation to hypoxia, thus promoting tumor progression (54, 55). Moreover, STC2 plays an important role in CRC progression and prognosis and could be a biomarker for survival prediction (55). However, there is little research about ANO1, ATP6V1B1, and HMCN2 in tumors, and it may be better to pay more attention to them.

Thereafter, we identified the biological processes and immune characteristics of HRGPI subgroups. The HRGPI-high group was presented as higher activity of the HIF-1 pathway, a higher score of angiogenesis process, and a higher infiltration level of immune cells and stromal cells than the HRGPI-low group, inclining to present more obvious hypoxia patterns than cluster B. The immune characteristics in the HRGPI-high group were similar to those in cluster B, while those in HRGPI-low were similar to those in cluster A, which indicated that our HRGPI could accurately evaluate and quantify the hypoxia-related characteristics of patients with COAD. We also found that the HRGPI-high group was mainly enriched in immune- and cancer-related pathways, such as cytokine–cytokine receptor interaction and Toll-like receptor signaling pathways. Moreover, the HRGPI-low group was mainly enriched in the metabolism-related pathways, including ascorbate metabolism, drug metabolism cytochrome P450, etc.

Integrated with clinical stages and molecular subtypes, HRGPI could distinguish different clinical and molecular subtypes of COAD. In terms of the clinical stages, there were more patients with advanced stages in the HRGPI-high group than in the HRGPI-low group. The clinical stage is the most critical prognostic index for patients with COAD, and an early stage indicates a better outcome (56). We also found that the proportions of CMS subtypes in HRGPI subgroups were different, and there were more patients classified into CMS1 and CMS4 and fewer samples classified into CMS2 and CMS3 in the HRGPI-high group than in the HRGPI-low group. According to Guinney et al., the CMS1 subtype is characterized by increased expression of genes related to a high-level immune infiltrate mainly composed of TH1 cells and cytotoxic T cells (CTLs), together with the activation of immune evasion pathways (22). CMS4 displays a worse prognosis containing OS and relapse-free survival, showing upregulation of genes in EMT, a high level of stromal infiltration, and activation of transforming growth factor (TGF-β) signaling, angiogenesis, and matrix remodeling pathways (22).

Interestingly, we uncovered that HRGPI might reflect different benefits from ICI therapy (anti-PD1 and anti-CTLA4). The HRGPI-high group had a higher TIDE score, which indicated a higher potential for immune evasion and a fewer benefit from ICI therapy. Meanwhile, higher T-cell exclusion scores and T-cell dysfunction scores were observed in the HRGPI-high group, suggesting a lower ICI response might be due to immune evasion *via* T-cell exclusion and T-cell dysfunction (23). To further investigate the prognostic value of HRGPI, the IMvigor210 (mUC) cohort was also used to verify the benefits of ICI in the two HRGPI groups. We found that HRGPI could differentiate outcomes in patients with COAD who received anti-PD-L1 therapy. Moreover, the patients with a lower HRGPI score are inclined to present a CR/PR status after ICI treatment and have a better OS. Hypoxia plays an essential role in preventing the effectiveness of ICI. Under hypoxia stress, tumor cells produce more adenosine and secret it in extracellular surroundings, resulting in the suppressor of T cells (57, 58). Moreover, HIF-1 is a vital inhibitor for the adaptive immune system (59). This was also consistent with our results above.

## Conclusion

In conclusion, different hypoxia-responding patterns present distinct biological processes, signaling pathways, and immune features. Under severe hypoxia status, the TME of COAD was complex and tended to present as an immune-excluded phenotype. HRGPI grouping has an advantage in distinguishing immune and molecular characteristics. Furthermore, the HRGPI could be an independent prognostic factor for COAD patients and an excellent predictive indicator for clinical response to ICI therapy.

## Data Availability Statement

The datasets presented in this study can be found in online repositories. The names of the repository/repositories and accession number(s) can be found in the article/**Supplementary Material**.

## Author Contributions

JR conceived and supervised the study. JR, SB, LC, YY, RL, YZ, XW, HK, ZF, GL, SZ, and ED analyzed data. JR and SB wrote the manuscript. JR and SB made manuscript revisions. All authors have read and approved the final version of this submission.

## Funding

This manuscript is supported by the Scientific and Technological Research Foundation of Shaan’xi Province (Juan Ren, 2020JM-368).

## Conflict of Interest

The authors declare that the research was conducted in the absence of any commercial or financial relationships that could be construed as a potential conflict of interest.

## Publisher’s Note

All claims expressed in this article are solely those of the authors and do not necessarily represent those of their affiliated organizations, or those of the publisher, the editors and the reviewers. Any product that may be evaluated in this article, or claim that may be made by its manufacturer, is not guaranteed or endorsed by the publisher.

## Supplementary Material

Supplementary Figure 1 Detailed results of survival analysis (Kaplan–Meier) for OS in meta-cohort.

Supplementary Figure 2 Detailed results of survival analysis (Kaplan–Meier) for OS in TCGA cohort.

Supplementary Figure 3 Detailed results of survival analysis (Kaplan–Meier) for OS in GEO cohort.

Supplementary Figure 4 Different biological processes between the HRGPI-high group and the HRGPI-low group.

Supplementary Figure 5 Detailed results of survival analysis (Kaplan–Meier) for PFS in meta-cohort.

Supplementary Figure 6 The distribution of HRGPI scores in cluster A-D.
