# Supplementary figures and images for: Exploration of Different Hypoxia Patterns and Construction of a Hypoxia-Related Gene Prognostic Index in Colorectal Cancer
Source: Front Immunol. 2022 May 30;13:853352. doi: 10.3389/fimmu.2022.853352 (PMC9196334; doi:10.3389/fimmu.2022.853352)

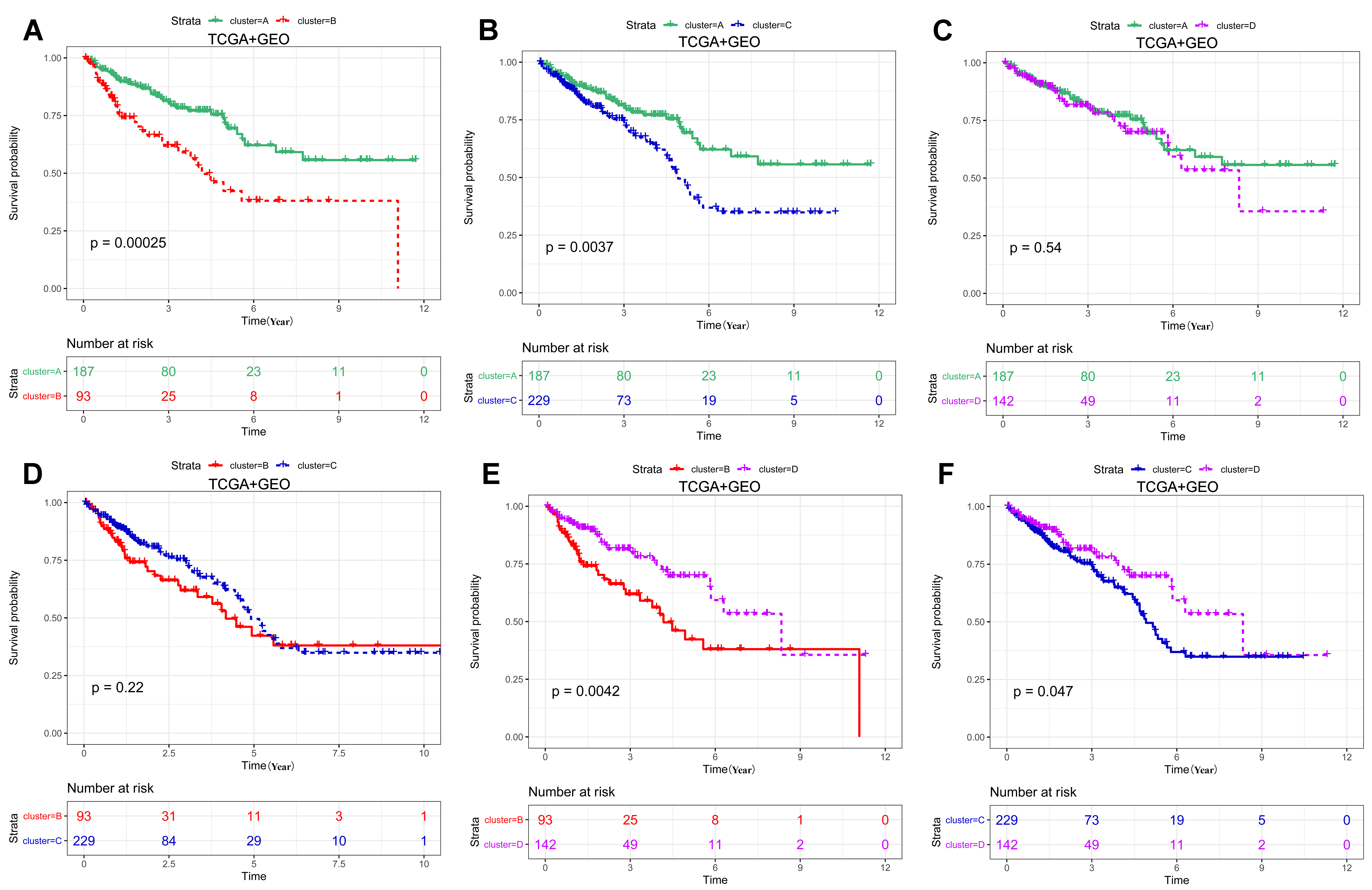

Supplement: Supplementary Figure 1 — Detailed results of survival analysis (Kaplan–Meier) for OS in meta-cohort. [file Image_1.tif]

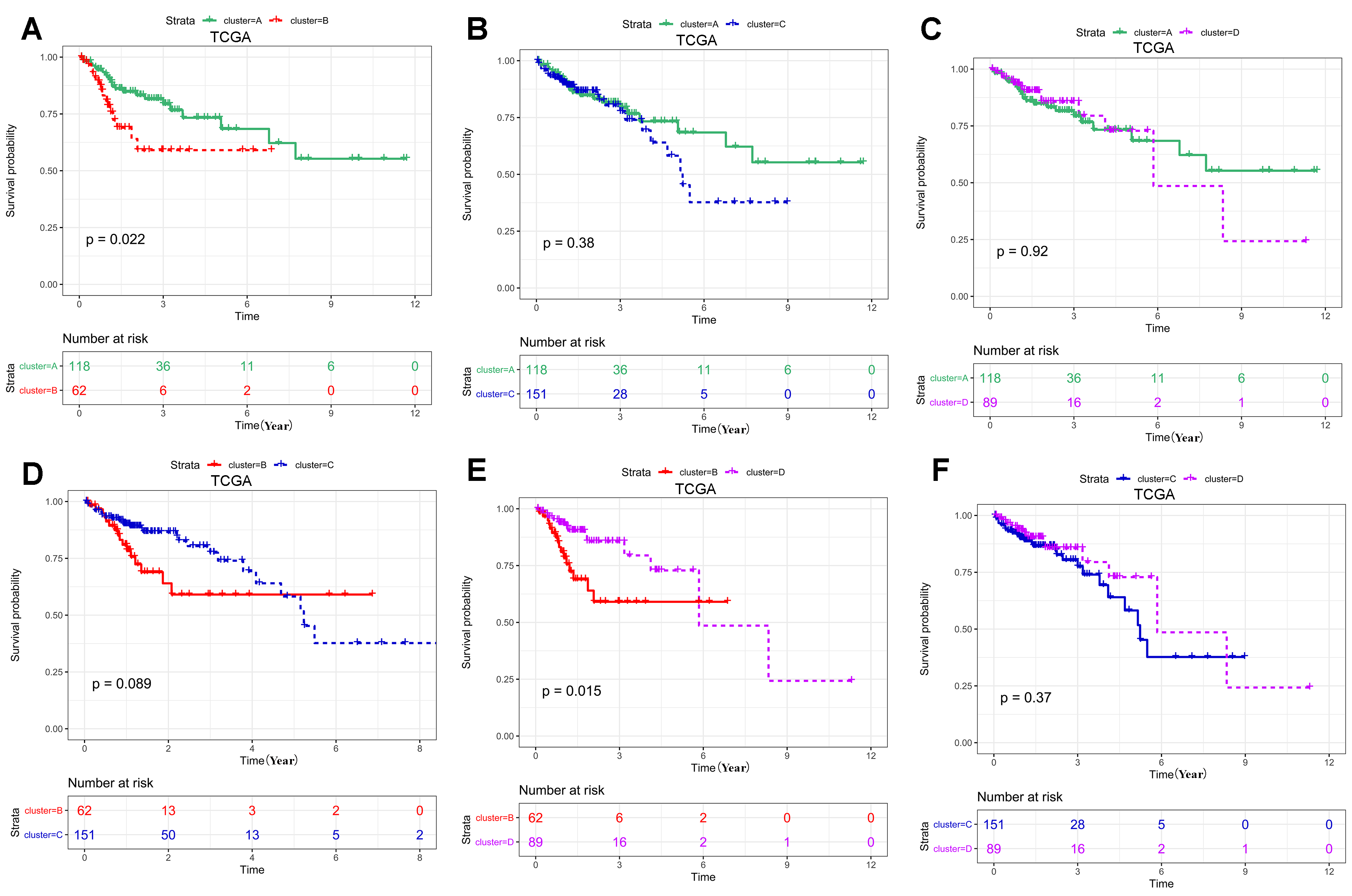

Supplement: Supplementary Figure 2 — Detailed results of survival analysis (Kaplan–Meier) for OS in TCGA cohort. [file Image_2.tif]

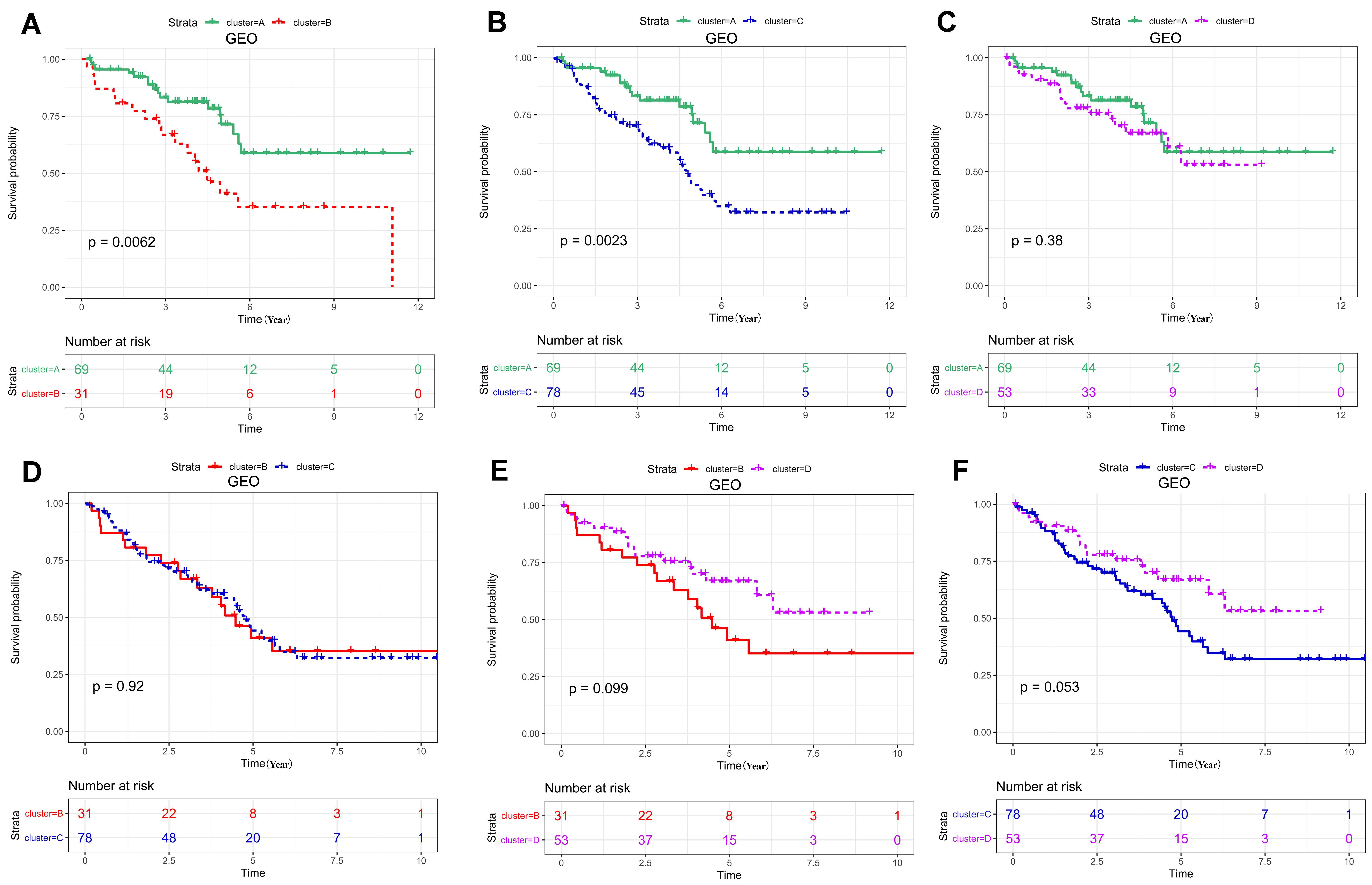

Supplement: Supplementary Figure 3 — Detailed results of survival analysis (Kaplan–Meier) for OS in GEO cohort. [file Image_3.tif]

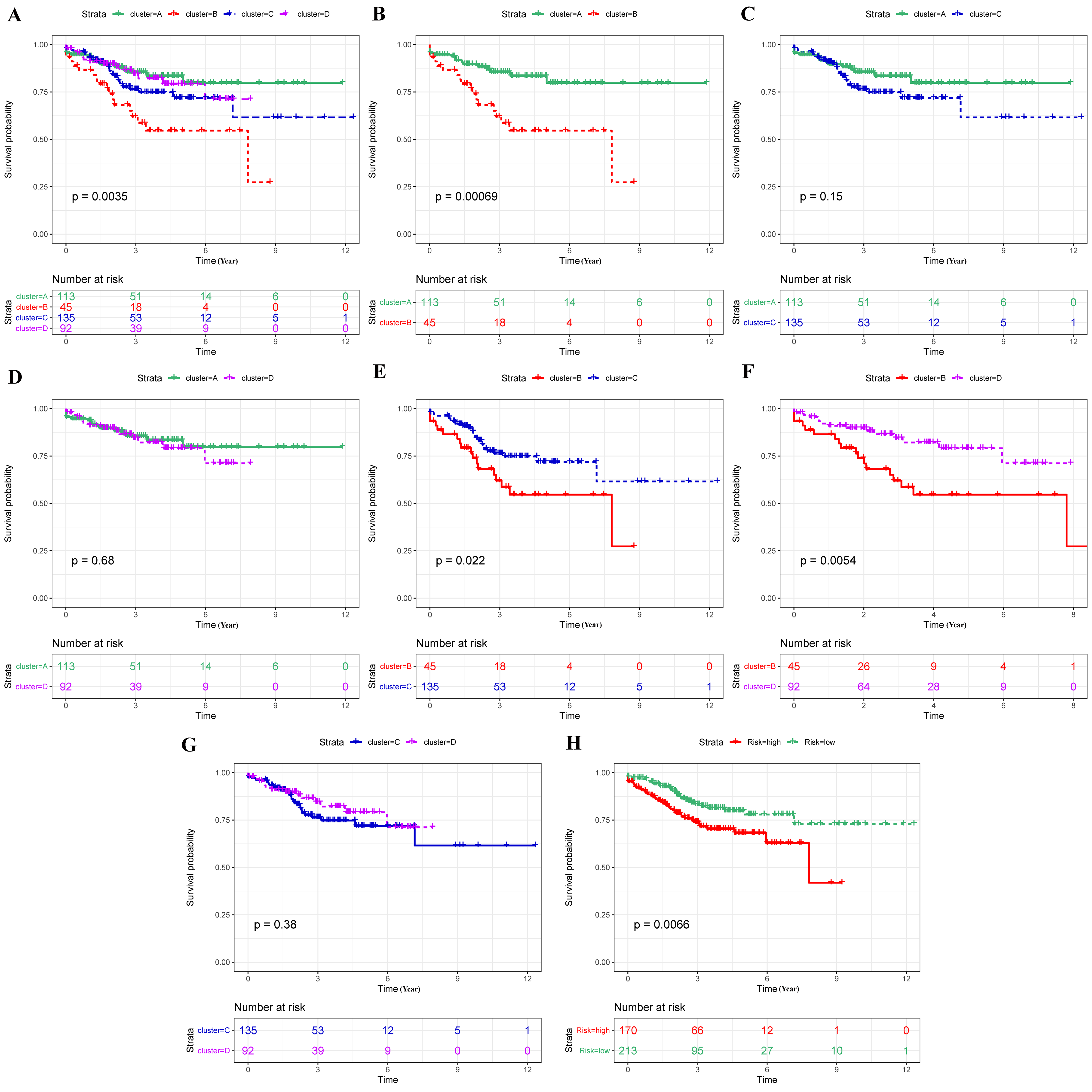

Supplement: Supplementary Figure 4 — Different biological processes between the HRGPI-high group and the HRGPI-low group. [file Image_4.tif]

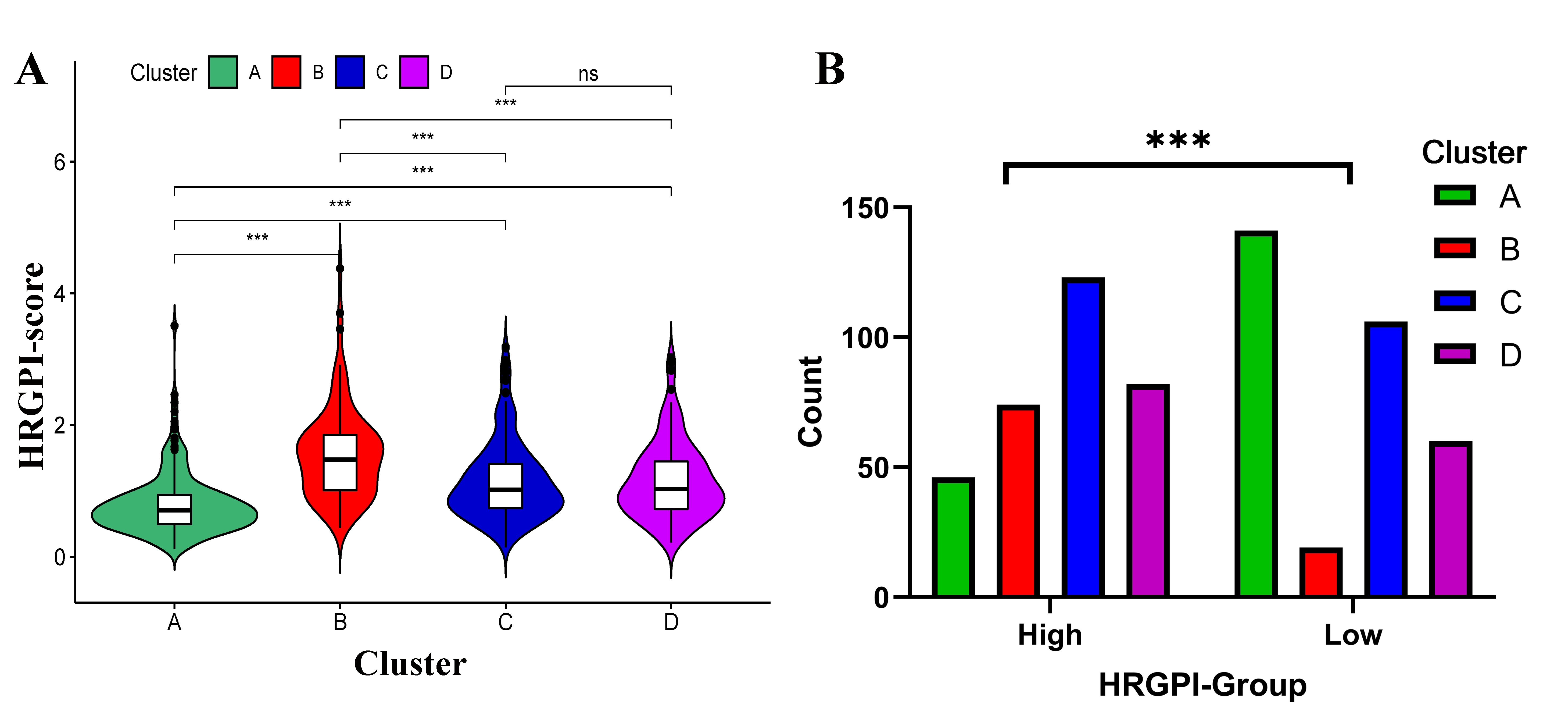

Supplement: Supplementary Figure 5 — Detailed results of survival analysis (Kaplan–Meier) for PFS in meta-cohort. [file Image_5.tif]

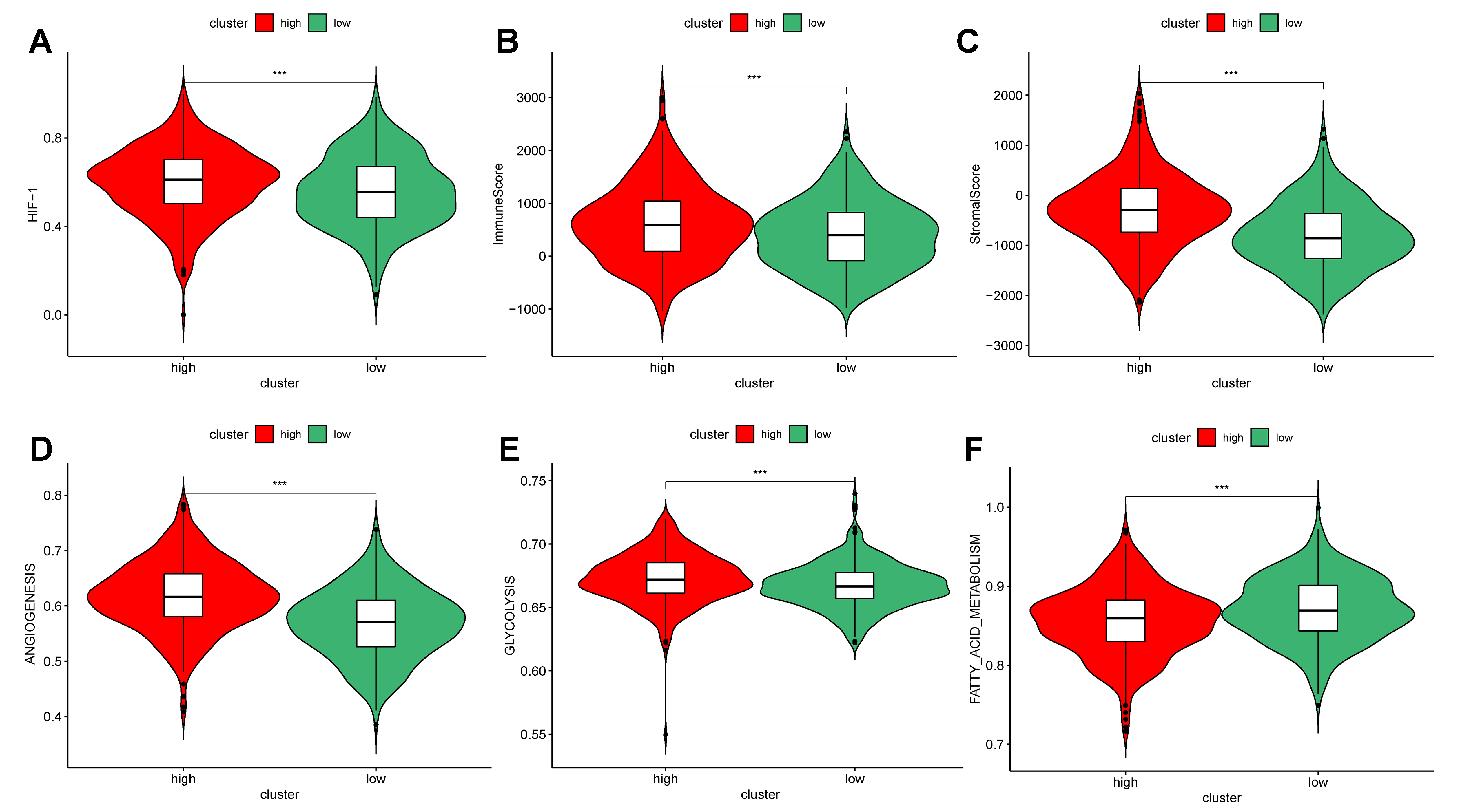

Supplement: Supplementary Figure 6 — The distribution of HRGPI scores in cluster A-D. [file Image_6.tif]
